# The Mechanical Adaptation of the Obstetric Forceps

**Published:** 1880-02

**Authors:** Philip Adolphus


### ARTICLE V.

### THE MECHANICAL ADAPTATION OF THE OBSTETRIC FORCEPS.

By PHILIP ADOLPHUS, M.D.

(During a joint discussion before the Chicago Medical Society, on Oct. 1, 1879, the following paper was read.)

I address you on the mechanical adaptation of the obstetric forceps. I understand this to apply to its mechanical adaptation to the obstetric canal of a woman in labor. Not the osseous pelvis merely, but the pelvis covered with soft parts, whose terminal outlet is not at the point of the coccyx, but at the anterior commissure of a greatly distended perineum—a distance of ten to twelve inches during labor.

The fundamental principles of midwifery are deduced from the correspondence between the fœtal dimensions and those of the pelvis.

The fœtal head, ovoidal in form, generally presents itself at the superior strait, with one of the extremities of an oblique diameter strongly flexed upon the trunk, so that its smallest diameter shall be parallel to the strait.

The central line traversed by the fœtus, from its entrance into the superior strait until its final exit from the vulva, whilst the patient is lying on the back, is a strongly marked curve, whose concavity is turned almost directly upward.

The contractions of the uterus, the abdominal muscles, and the resistance of the perineum, propel the child through the obstetric canal.

The inclined planes are supposed to play an important part in the mechanism of labor, for their direction has an immediate influence upon the movements which the head of the fœtus performs in the excavation.

The head, when arrested during labor by local conditions or general complications on the part of the mother, requires delivery by an instrument which must be adapted to the form and size of the child's head as well as to the anatomical conformation of the pelvis and soft parts of the parturient canal. The straight forceps, having merely a cephalic curve, are only employed when the head is within the pelvis ; it has short handles and is without shanks ; therefore, it is a feeble instrument, either as a tractor, lever or compressor, being only useful when very little tractile power is required. But the long, double-curved forceps, having both the cephalic and pelvic curves, with lengthened blades and shanks, which can traverse the pelvis, from its entrance into the superior strait until its exit from the vulva, without inflicting injuries to the fœtus and the mother, is an instrument which, by its tractile, compressive and leverage powers, can and does save many a fœtus from perforation. It is the only instrument that joins mechanical adaptation to all portions of the parturient canal with power, and should be the only one adopted by the busy practitioner as worthy of confidence in all cases where forceps are indicated. It is a tractor, secondarily a compressor ; occasionally limited lever-like movements are admissible, but rotation must not be performed with it, for the fœtus will rotate spontaneously within its blades during traction, owing to the anterior and posterior planes on either side of the cavity and the resistance of the floor of the pelvis.

The governing factors in the use of the long forceps are "anatomical knowledge, mechanical skill and common sense ;" from these and experience the following rules have been deduced:

The position of the head does not determine the position of the blades ; but the position of the blades is always determined by the anatomy of the mother.

Therefore, the forceps must be applied along the sides of the pelvis, and its pelvic curves must correspond to its curved axis.

Its introduction is governed by the direction of the obstetrical canal, the globular head of the child, and the cranial and pelvic curves of the instrument.

"In its introduction and removal it follows the circle of which it forms a part ;" for it will always be coincident with the obstetric canal, from the superior strait to its outlet.

It is fit that the exact position of the head should be known, but such knowledge is not essential to its safe extraction; on the contrary it is not correct to apply the forceps to the sides of the fœtal head when its position is oblique or transverse, for if its pelvic curves are twisted, injury must be inflicted to the mother.

